# Geospatial techniques for monitoring and mitigating climate change and its effects on human health

**DOI:** 10.1186/s12942-023-00324-9

**Published:** 2023-01-27

**Authors:** Maged N. Kamel Boulos, John P. Wilson

**Affiliations:** 1 Co-Chair, WG III/9 Geospatial Environment and Health Analytics, ISPRS Technical Commission III, 30167 Hannover, Germany; 2 School of Medicine, University of Lisbon, 1649-028 Lisbon, Portugal; 3 Spatial Sciences Institute, Dornsife College of Letters, Arts and Sciences, University of Southern California, Los Angeles, CA 90089-0374 USA

**Keywords:** Climate change, Human-induced warming, Geospatial data, Geographic information systems (GIS), Remote sensing, Human health, Public health, Healthcare

## Abstract

This article begins by briefly examining the multitude of ways in which climate and climate change affect human health and wellbeing. It then proceeds to present a quick overview of how geospatial data, methods and tools are playing key roles in the measurement, analysis and modelling of climate change and its effects on human health. Geospatial techniques are proving indispensable for making more accurate assessments and estimates, predicting future trends more reliably, and devising more optimised climate change adaptation and mitigation plans.

## Introduction

Human-induced warming (climate change) is a major public health concern due to its devastating effects on nature and human health. This article begins by examining how climate change is impacting our health and wellbeing. It then discusses the key roles played by geospatial techniques in measuring, analysing, modelling, and mitigating climate change and its effects on our lives and future generations.

### The impact of climate change on human health

The main manifestations of climate change, namely more extreme weather, increased carbon dioxide levels, and rising temperatures and sea levels, result in severe weather events, extreme heat, environmental degradation, air pollution, increasing allergens, food supply and water quality issues, and changes in disease vector ecology, all of which have significant adverse effects on human health (Table 1) [1] and on healthcare services [2].

**Table 1:** Effects of climate change on human health (examples)

Climate change aspect (exposures and pathways)	Human health impact (examples of increased adverse health outcomes and diseases)
Severe weather factors	Injuries, fatalities and negative mental health impacts (e.g., [3])
Extreme heat	Heat-related illness and death (e.g., [4, 5]), cardiovascular disease and failure (e.g., [6–8]), kidney disease (e.g., [9]) and adverse reproductive effects (e.g., [10])
Environmental degradation	Forced migration, civil conflict and negative mental health impacts (e.g., [3])
Air pollution and increasing allergens	Respiratory allergies, asthma (e.g., [11]), cardiovascular disease (e.g., [8]) and neurological conditions (e.g., [12])
Water and food supply impacts (e.g., [13]), including water quality issues	Malnutrition (e.g., [14]), diarrhoeal disease, cholera [15], cryptosporidiosis, campylobacter (e.g., [13]), leptospirosis and harmful algal blooms
Changes in disease vector ecology	Malaria (e.g., [16, 17], dengue (e.g., [18]), encephalitis, hantavirus, Rift Valley fever, Lyme disease, chikungunya and West Nile virus (e.g., [19])

Interestingly, while climate change unarguably has major effects on human disease and clinical practice, the reverse is also true. Our healthcare systems and treatment of certain conditions can sometimes negatively impact human-induced climate change. Young et al. give the example of haemodialysis, which although lifesaving, is associated with significant water and energy consumption per session and with large clinical wastes. They conclude that there is an urgent need to develop more climate-responsible methods for managing kidney disease [9].

Unfavourable environmental factors kill around 13 million people worldwide every year [20]. Furthermore, according to some estimates, climate change will lead to approximately 250,000 additional deaths per year between 2030 and 2050 as a result of heat stress, malaria, malnutrition and diarrhoea. Many of these deaths can be avoided by planning and implementing appropriate transformational solutions. For example, current food production and distribution systems generate a third of greenhouse gas emissions. Switching to more sustainable food supply systems would mitigate climate impacts whilst allowing for more nutritious diets, which could prevent about 11 million premature deaths a year [21].

However, accurately quantifying the exact impacts of climate change (and any corresponding mitigatory solutions) on human health remains a challenge. The interplay between human-induced warming, exposed populations and the local healthcare systems serving them is quite complex (and ultimately determines observed health outcomes), but scientific advances in various fields, including geospatial technologies, are increasingly allowing us to make more accurate morbidity and mortality estimates, predict future trends more reliably, and devise more optimised adaptation and mitigation plans [15, 17, 21–25].

### Geospatial techniques for climate change

Developments in geographic information science (GISc) have transformed how researchers in a variety of fields gather and analyse information about the world around us. Scientists gain valuable insights in areas such as geology and meteorology by gathering remote sensed data and exploring these data with geospatial tools [26, 27]. The perspectives offered by geographic information science and the accompanying geospatial technologies and methods span multiple scales and domains and as such, are particularly helpful in studying global climate change [28].

Geographic information science combines two major threads which fit nicely within the pluralistic, complex, and multi-paradigmatic vision of GISc envisaged by Blaschke and Merschdorf [29]. The first thread, which took shape quickly following the establishment of the US National Center for Geographic Information and Analysis in 1988, focuses our attention on the need to represent, measure and manipulate information on space and time in precise and reproducible ways and remains important to this day [30].

The second thread, which challenges the aforementioned view of the world, starts with the spatial "turn" that has swept through the sciences, social sciences and the humanities during the past four decades [31, 32], and sets up a clash between the rigorously precise measurements that characterise the first thread and the uncertainty and ambiguity that pervades life and place in the second thread [33]. The latter relies on a new epistemology, one that is nonlinear, fluid and reflexive, and focuses on the use of space and time to reveal the complex and contingent context of processes and events with and across space and time.

This second thread has enormous potential for understanding nearly every aspect of the human condition, including the connections between climate change, health and place. Susan Kemp has written that "setting place outside of history flattens human experience, reducing it to a single plane of the present, and obscuring the deep-rooted social, political, and economic mechanisms at the core of health disparities" [34]. The latter is important because this view means that historical patterns of social and environmental risk will help to shape the impact of climate change on human health and wellbeing, and ensure that inequalities in health (and life generally) persist over multiple generations (e.g., [35]). Some of these same issues lie at the heart of work by Marek et al. [36] which attempts to map health‑promoting and health‑constraining environments in New Zealand.

The remainder of this article is cast in two parts. The first focuses on the use of geographic data and methods for documenting climate change and its impacts. The second shows an example of how these same data and methods can be deployed to help mitigate heat impacts among vulnerable urban communities.

## The role of geospatial data, methods and tools in the measurement, analysis and modelling of climate change and its effects

According to NASA (US National Aeronautics and Space Administration), the Earth's average surface temperature has increased by approximately 0.9 °C since the late nineteenth century. Scientists attribute problems such as extreme weather events, rising sea levels and diminished ice sheets and glaciers to the emission of carbon dioxide and other greenhouse gases into the atmosphere. The Fourth National Climate Assessment, released by the US Global Change Research Program in November 2017, predicted long-term consequences such as:

- Changing patterns of precipitation and severe weather events;
- A decline in crop production;
- Diminished populations of underwater creatures due to ocean acidification;
- Worsening air quality;
- Greater incidence of food and waterborne diseases; and
- An increase in heat-related deaths in the US [37].

Using GIS (geographic information systems) methods and software, experts can closely monitor these dangers. Robust geospatial data and detailed visualisations offer guidance for organisations and government agencies as they plan for the challenges ahead. By bringing together GIS and climate change studies, spatial problem-solvers can seize opportunities to make a difference in the lives of future generations (e.g., [22]).

Climate scientists gather vast amounts of data to track environmental problems and investigate their causes. Spatial reasoning is crucial for analysing and synthesising findings from sources including in-situ sensors and satellite imagery. Researchers draw on geographic information to reveal how the planet has changed through the years, predict the transformations that are yet to come, and communicate what they learn to policymakers and the public (e.g., [38–41]).

Continuing advances in GIS technology have established mapping as a crucial means of identifying connections between the state of the climate and other areas of concern. Open-source databases allow for unprecedented collections of spatial information, and high-speed data processing reveals changing conditions in real time (e.g., [42–44]).

Some of the ways that researchers are using GIS for information on climate change include:

- Locating areas where temperatures are particularly high in comparison to regional or global averages;
- Discovering how natural atmospheric processes might affect global warming;
- Creating models to show how a warming climate might impact the ecology of various regions;
- Examining the relevance of shifts in land cover, such as the removal of trees, to climate change; and
- Visualising multiple factors with the potential to affect crop growth, industry, wildlife and human wellbeing.

Geospatial tools offer perspective on evolving weather patterns, rising sea levels and growing risks to human health. Informative visualisations show these developments in ways that scientists and non-experts alike can understand. In turn, local governments, non-profits and other organisations apply what they learn from geographic models to set strategy and make informed decisions.

Scientists have linked the warming climate to extreme weather such as floods, droughts, heat waves and hurricanes. Evidence suggests that increasing global temperatures may make these events more frequent and severe. Geospatial methods improve our understanding of how climate change affects weather patterns and can warn communities of dangers to infrastructure, residents and property.

For example, a study for the Pennsylvania Department of Transportation prepared by Michael Baker International in 2017 set out to examine the state's history of flooding, construct a framework to account for the climate's impact on future storms, and predict problems that might occur due to extreme weather. The research team used GIS to analyse relevant information, such as historical precipitation data and risk assessment scores for roads and bridges, and generate visualisations [45].

When extreme weather does strike, geospatial intelligence provides support for first responders and recovery workers. Hazard maps based on remote sensing data and satellite imagery keep government officials informed about current conditions and what areas have the greatest need of urgent attention (e.g., [46]). Response teams provide updates and photos from the ground, leading to efficient and effective crisis management (e.g., [47]). After a major storm or fire is under control, geographic information guides utility companies as they restore service and assists in the planning processes for replanting crops or rebuilding structures.

## Using GIS to connect need and opportunity: an example of how GIS is helping mitigate heat impacts among vulnerable urban communities

The pervasive nature of climate change coupled with the unique characteristics of specific regions, cities and neighbourhoods will require well-tailored, place-based interventions to mitigate its effects. The example below shows how some of these same GIS methods and geospatial data can be deployed to help mitigate heat impacts among vulnerable communities in large cities.

The example is drawn from an ongoing project that endeavours to describe the current urban canopy and opportunities to expand this canopy moving forward to help mitigate the heat impacts that will accompany a warming climate in five low-income communities of colour in Los Angeles County. The choice of the five communities—Boyle Heights, City Terrace, El Sereno, Lincoln Heights and University Park—was intentional. These areas are home to more people than trees. In addition, many of their 250,899 residents have lived in these neighbourhoods for > 10 years notwithstanding median household incomes of USD $45,540 and the presence of just 109,693 trees. There are also substantial numbers of children and older adults, 3.6 persons per household, and one in six households lacks an automobile. Many of the homes and apartments, and the lots themselves, are modest in size and given poor ventilation and few air conditioning options, they offer few opportunities for handling the heat on days with air temperatures > 32 °C, which are expected to triple in number by 2050 [48].

The opportunities and accompanying challenges for building a large and vibrant canopy in communities like these are enormous. A recent national (US) study of 5723 communities showed how low-income neighbourhoods with large numbers of people of colour have experienced longstanding inequities in terms of green cover that date back to the redlining era (the term 'redlining' was coined in the 1960s to describe discriminatory banking practice that classified certain neighbourhoods as 'risky' or not worthy of investment based on the racial makeup of their residents) [49]. A recent local study indicates that these inequities are likely growing in the Los Angeles Basin given steeper declines in tree cover on single family home lots in these types of neighbourhoods compared to high income areas (Pacific Palisades, Palos Verdes, and Pasadena) during the period 2000–2010 [50].

This said, there is a need to acquire and use geospatial data in order to plant a larger and more equitable tree canopy across the City of Los Angeles and the surrounding communities. The same aforementioned GIS methods and geospatial data can be used to predict pedestrian traffic and existing tree canopy around elementary schools. The three-step workflow uses a street network, the locations of residential units, the gates through which children enter or leave school campuses, the street trees on the routes to and from the schools, the enrolment area boundaries and the total numbers of students enrolled in each school. The first step takes the street centrelines and residential households and builds pedestrian traffic counts over 100 m intervals to and from the target elementary school. The second step takes the street tree information and uses this with the predicted pedestrian counts to identify the streets or parts of streets with high traffic and little or no tree canopy. The third step entails taking both a broader and deeper look—broad in the sense that we can compare results across elementary school enrolment areas in multiple neighbourhoods to identify the streets with the largest discrepancies between pedestrian traffic and tree canopy, and deeper because of the need to examine extenuating circumstances, such as overhead utility lines, the need for concrete cuts to plant new trees and existing municipal codes that may limit tree planting near street intersections and transit stops.

**Fig. 1:**
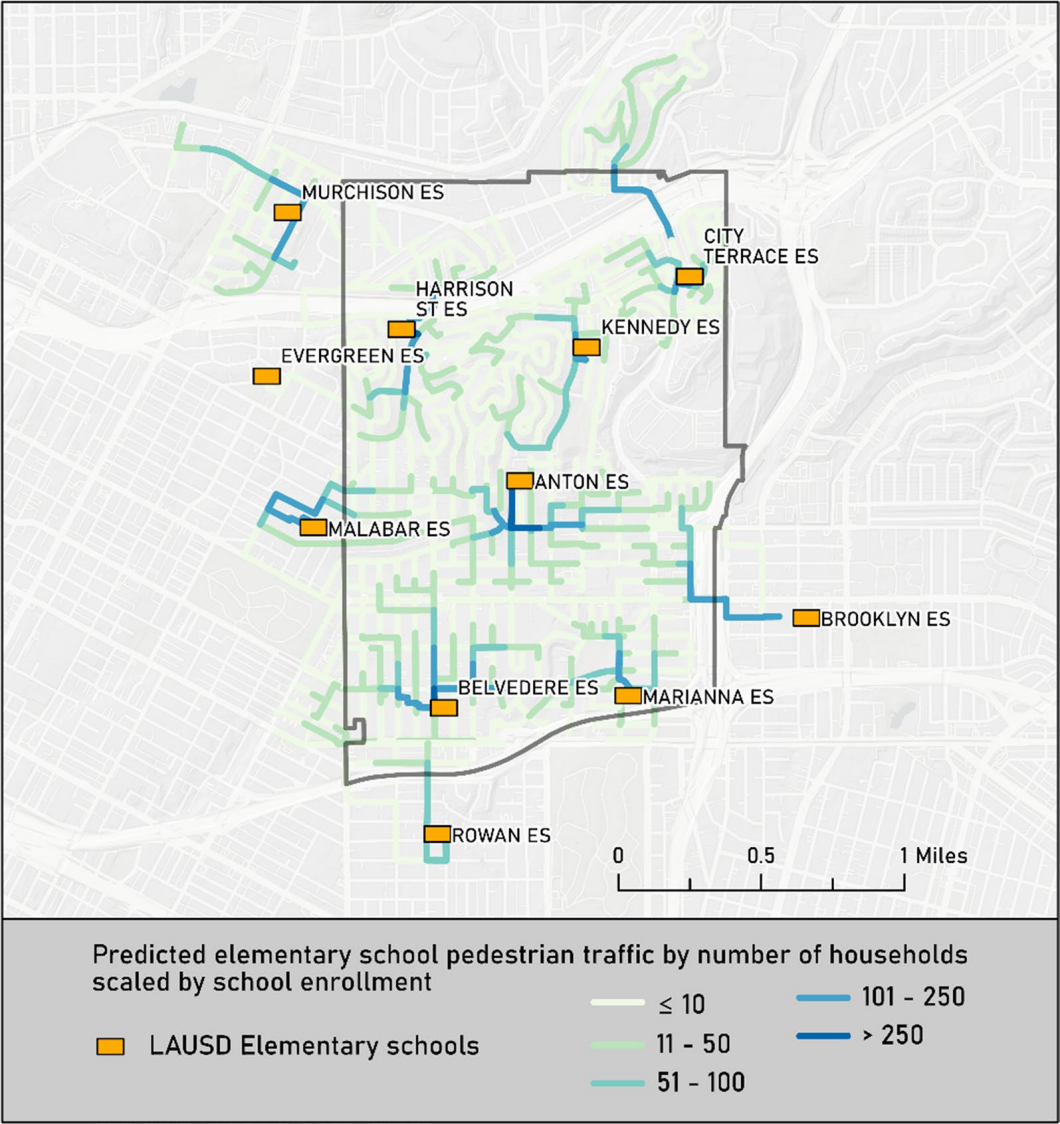
Predicted elementary school pedestrian traffic by number of households scaled by school enrolment in the City Terrace, California neighbourhood

The map reproduced in Fig. 1 shows the predicted pedestrian traffic for elementary schools that serve students who reside in City Terrace, a neighbourhood in unincorporated Los Angeles County located immediately east of the Boyle Heights neighbourhood referred to earlier. The streets coloured dark blue are traversed by parents and/or children from > 250 households (in this example), while lighter colours refer to streets traversed by fewer elementary school children and their parents or caregivers. This method was validated using observed pedestrian traffic counts for one elementary school provided by the Los Angeles County SRTS (Safe Routes to School) programme.

## Conclusions

Human-induced warming is affecting our health and wellbeing in a multitude of ways, causing considerable morbidity and mortality, most of which can be avoided through appropriate mitigatory actions and transformational solutions. Geospatial data, methods and tools are playing key roles in the measurement, analysis and modelling of climate change and its effects on human health. They are proving indispensable for making more accurate assessments, estimates and forecasts, and for devising more optimised climate change adaptation and mitigation plans.

## Data Availability

Data sharing is not applicable to this article, as no datasets were generated or analysed for the current paper.
